# Quantification of gait parameters in freely walking rodents

**DOI:** 10.1186/s12915-015-0154-0

**Published:** 2015-07-22

**Authors:** César S. Mendes, Imre Bartos, Zsuzsanna Márka, Turgay Akay, Szabolcs Márka, Richard S. Mann

**Affiliations:** Department of Biochemistry and Molecular Biophysics, Columbia University, 701 West 168th Street, HHSC 1104, New York, NY 10032 USA; Department of Physics, Columbia University, 538 West 120th Street, 1009 Pupin, MC 5229, Box 29, New York, NY 10027 USA; Department of Medical Neuroscience, Dalhousie University, Halifax, NS B3H 4R2 Canada; Current address: CEDOC, Faculdade de Ciências Médicas, Universidade Nova de Lisboa, Lisboa, 1169-056 Portugal

## Abstract

**Background:**

Qualitative and quantitative measurements of motor performance are essential for characterizing perturbations of motor systems. Although several methods exist for analyzing specific motor tasks, few behavioral assays are readily available to researchers that provide a complete set of kinematic parameters in rodents.

**Results:**

Here we present MouseWalker, an integrated hardware and software system that provides a comprehensive and quantitative description of kinematic features in freely walking rodents. Footprints are visualized with high spatial and temporal resolution by a non-invasive optical touch sensor coupled to high-speed imaging. A freely available and open-source software package tracks footprints and body features to generate a comprehensive description of many locomotion features, including static parameters such as footprint position and stance patterns and dynamic parameters, such as step and swing cycle duration, and inter-leg coordination. Using this method, we describe walking by wild-type mice including several previously undescribed parameters. For example, we demonstrate that footprint touchdown occurs instantaneously by the entire paw with no obvious rostral–caudal or lateral–medial bias.

**Conclusions:**

The readily available MouseWalker system and the large set of readouts it generates greatly increases the currently available toolkit for the analysis of wild type and aberrant locomotion in rodents.

**Electronic supplementary material:**

The online version of this article (doi:10.1186/s12915-015-0154-0) contains supplementary material, which is available to authorized users.

## Background

Understanding the neuronal mechanisms that control motor behavior, such as locomotion, is one of the major challenges in neuroscience research. For this purpose, mice have become an important animal model mainly due to the recent advances in mouse genetics that allow the precise manipulation of the neuronal networks that underlie motor behavior [1, 2]. Further, these advances have resulted in a large number of mutant mice lines that model human diseases that affect motor behavior [3]. These advances also increase the importance of developing assays that measure motor behavior in quantitative and objective ways.

A large number of assays have been developed that allow indirect behavioral profiling of motor deficits either by manual qualitative assessments, the automatic quantification of motor activity in an open arena [4], or motor performance tests scoring the execution of a specific motor task. Some examples are measuring the time and number of failures an animal takes to cross a narrow beam [5]; the rotarod test, which measures the time an animal can remain in a rotating cylinder [6]; reaching tests, which measure manual dexterity while an animal tries to retrieve food pellets [7]; or the speed and distance traveled in a stationary running wheel [8]. Although these behavioral tests provide readouts for general motor performance, they reflect a combination of multiple deficits that cannot be teased apart post hoc and, in addition, do not provide specific information about locomotor coordination.

To analyze walking, several assays have been developed. For example, by placing reflective markers at leg joints, Leblond et al. measured angular variations of leg joints during walking in a treadmill of wild-type and spinalized mice allowing a complete reconstruction of the step cycle [9]. The same preparation can also be combined with simultaneous electromyographic recordings [9–12], which provide a correlation between muscle activation and leg movement. Although informative, this approach remains invasive and laborious while focusing on only one leg, thus precluding the extraction of additional gait features. Gait analysis is frequently achieved using the footprint test, in which the paws are coated with ink and the animal is allowed to walk over a sheet of white paper to generate a footprint pattern [13]. These patterns can be further analyzed for several static step parameters including stride length, feet support or footprint spread. However, this test fails to provide dynamic parameters of locomotion such as the duration of the step cycle or inter-leg coordination. Such parameters could be acquired by recording videos of rodents as they walk on a transparent surface, followed by a frame-by-frame analysis. However, this is a very labor-intensive approach and prone to errors due to the ambiguous transition between the swing and stance phases. To overcome this obstacle, Hamers and colleagues developed a quantitative gait analysis assay using an optical touch sensor based on frustrated total internal reflection (fTIR), which records and tracks the footprints of rodents as they move in a walkway [14, 15]. This approach has the additional advantage of not depending on any markers within the animal’s body that could interfere with the locomotion pattern. This method was later made available commercially [16, 17]. However, the associated analysis software packages only monitor footprints and do not have the flexibility to allow users to extract additional and unanticipated metrics. Moreover, these systems are very costly, deterring individual laboratories from acquiring them.

Sophisticated machine vision algorithms that provide increased temporal and spatial resolution have also been developed (for example, [18–20]). These methods allow the extraction of detailed data sets and provide a powerful, unbiased and quantitative description of animal behavior. However, they lack the resolution to provide a quantitative measure of gait and other kinematic features.

To accommodate the need for an improved method to analyze locomotion in the mouse, we describe a simplified and inexpensive fTIR setup combined with an open-source and user-friendly software package. Using this system, which we called MouseWalker, we extracted a large set of kinematic parameters from freely walking wild-type animals, including step patterns, footprint positioning, inter-leg coordination, and footprint contact parameters.

## Results

### MouseWalker

**Fig. 1:**
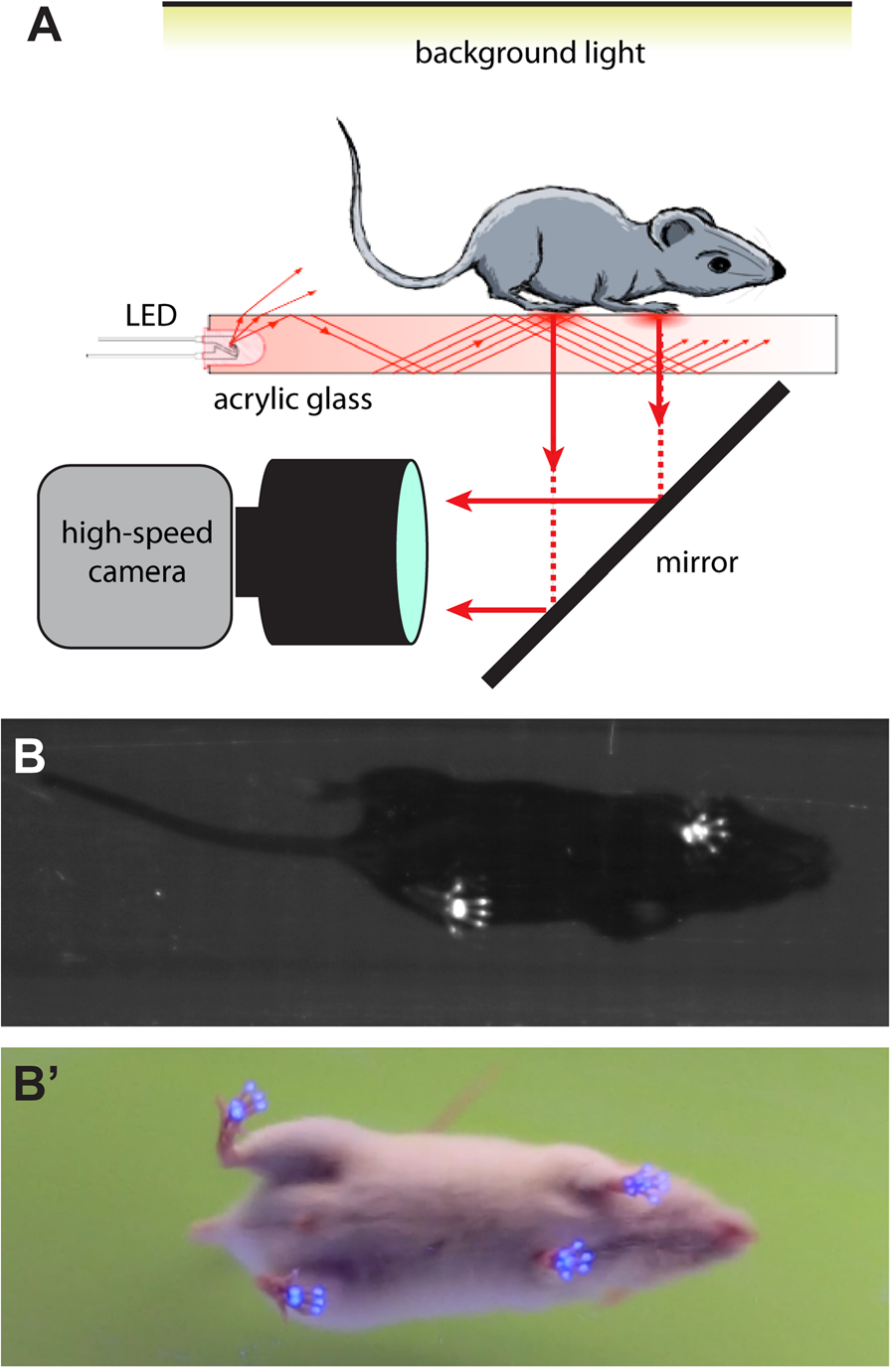
Optical footprint detection system. **a** Schematic of the fTIR effect. LED light sources are located at the edges of a piece of acrylic glass and light propagates within the glass via internal reflection. Footprints disrupt this optical effect leading to the light scattering, which is detected by a high-speed camera. Single frame of an fTIR video in black and white (**b**) and color (**b'**). The fTIR effect is visible while the legs are in contact with the acrylic glass surface during the stance phase. Background light allows detection of body contour

We previously described an approach to track and quantify kinematic properties of untethered walking fruit flies [21]. Using this approach we quantitatively described the walking behavior of wild-type and genetically modified animals [21, 22]. This method is based on the reflection of light within a transparent material through an optical effect termed total internal reflection [23, 24]. Foot contacts disrupt this effect causing fTIR, which generates scattered light that can be detected by a high-speed video camera (Fig. 1a). We built a simple walking apparatus from readily available and inexpensive supplies (Additional file 1: Figure S1), mostly precut acrylic glass and aluminum components (see Additional file 1: Figure S1, Additional file 2: Figure S2, Additional file 3: Figure S3, Additional file 4: Figure S4, Additional file 5: Figure S5 and “Methods” for details), in which the rodents can walk freely down a narrow corridor. The floor is of acrylic glass surrounded by LED lights, thus producing a touch sensor (Additional file 2: Figure S2). Empirically, we found that an acrylic glass surface resulted in a better fTIR signal-to-noise ratio compared to glass, possibly due to a rougher surface thus allowing more contact between the animal’s paws and the walking surface. Although acrylic glass scratches more easily than glass, thus interfering with the fITR signal and subsequent tracking, it can be easily replaced. A light box positioned above the walking apparatus allows the outline of the mouse body to be visualized as the animal moves along the walkway (Additional file 5: Figure S5). Finally, a mirror placed at 45° below the walking surface reflects the fTIR signal and body outline, allowing them to be captured by a high-speed camera (Fig. 1a and Additional file 4: Figure S4 and Additional file 6: Video S1). Depending on the type of camera available and color of the LEDs and light box, the setup can generate monochromatic or color videos (Fig. 1b).

**Fig. 2:**
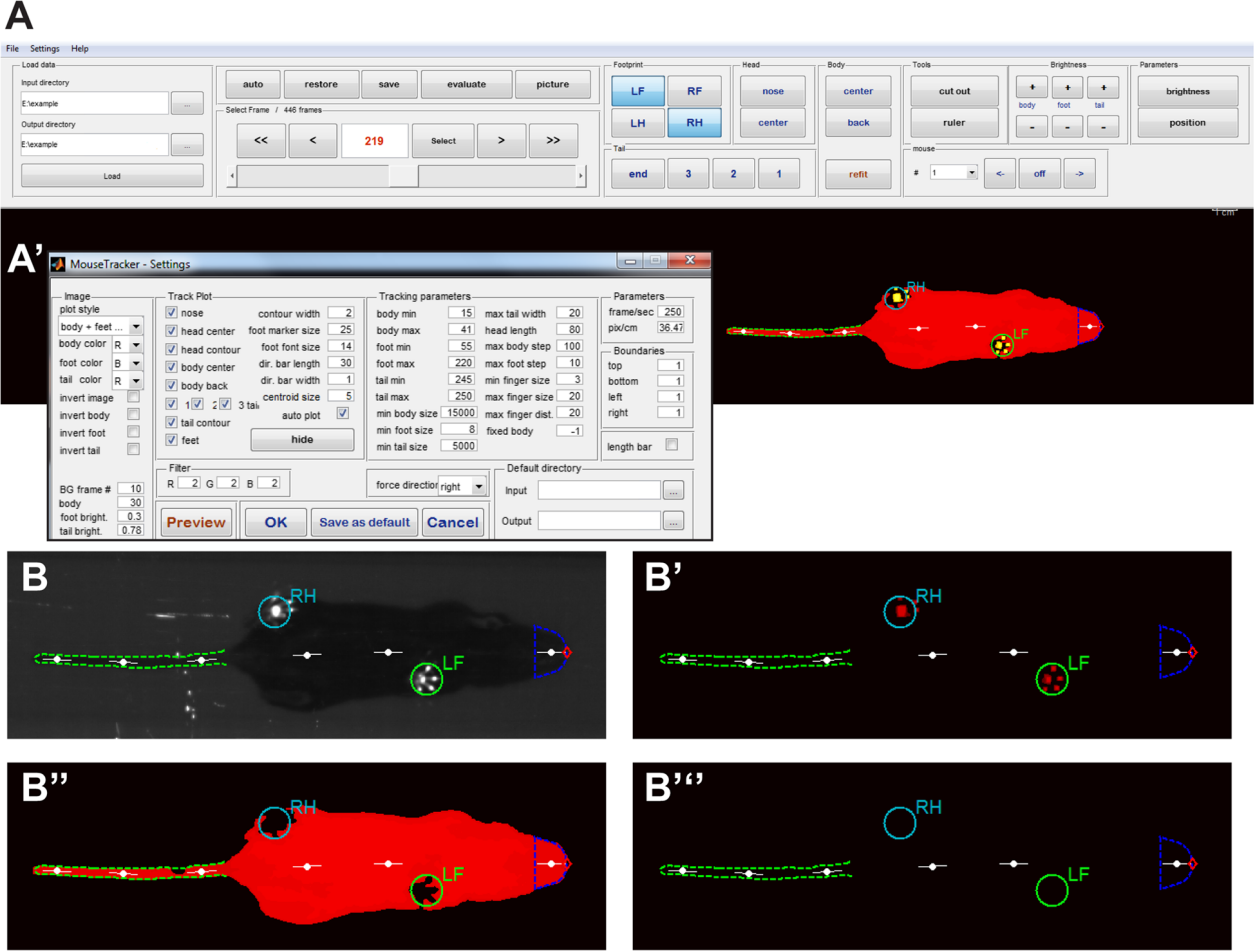
MouseWalker software interface. **a** Program layout. Videos are loaded, automatically tracked, and edited in a single window. **a**' Settings window provides detection and display options. Visualization options: tracking can be visualized simultaneously with the original video (**b**), with only the footprints (**b'**), with only the body contour (**b''**), or none of these (**b'''**)

Recorded videos are loaded into a program developed specifically for this assay (Fig. 2a'). The MouseWalker software was written in MATLAB and compiled as a standalone program, which analyzes the sequence of images from the videos by registering the position of the body, tail, and each footprint (Additional file 7: Video S2). Each video is loaded into the graphics user interface (GUI) of the program where the auto-tracking feature can identify each footprint, body contour, and position of the tail, with an accuracy >90 %, depending on the acquisition conditions and settings. Footprints and body contours are identified based on pixel intensity thresholds defined by the user in a dedicated settings window, which can be determined in a few minutes by an experienced user with the help of a preview section or by auto-tracking for a few frames (Fig. 2a'). Optimal settings can be stored for subsequent movies. Importantly, since the program can discriminate between red, green, and blue inputs, body elements can be identified based on color (Fig. 1b'). Subsequently, the user can manually edit any mislabeled footprints or body features if necessary. In addition, the user can toggle between different visualization modes (such as the unprocessed image, tracked body, or footprints; Fig. 2b and Additional file 7: Video S2, Additional file 8: Video S3, Additional file 9: Video S4), which help in setting the parameters and accurately editing the video. These are particularly important in identifying the footprint contacts and body. Most importantly, the user interface allows the generation of a set of output files (see “Methods” for a complete list), including an Excel file containing >20 quantifiable parameters (Additional file 10: Table S1) and an annotated video (Additional file 7: Video S2, Additional file 8: Video S3, Additional file 9: Video S4). The system is flexible in that add-on scripts can be written to extract additional parameters from the MATLAB tracks or Excel files.

### Walking by wild-type mice

As a proof of principle, we examined the walking behavior of a laboratory wild-type C57BL/6J strain moving across an 80-cm-long walkway. We collected 16 videos from four animals and analyzed the data as they moved continuously in the center 50 cm of the walkway.

**Fig. 3:**
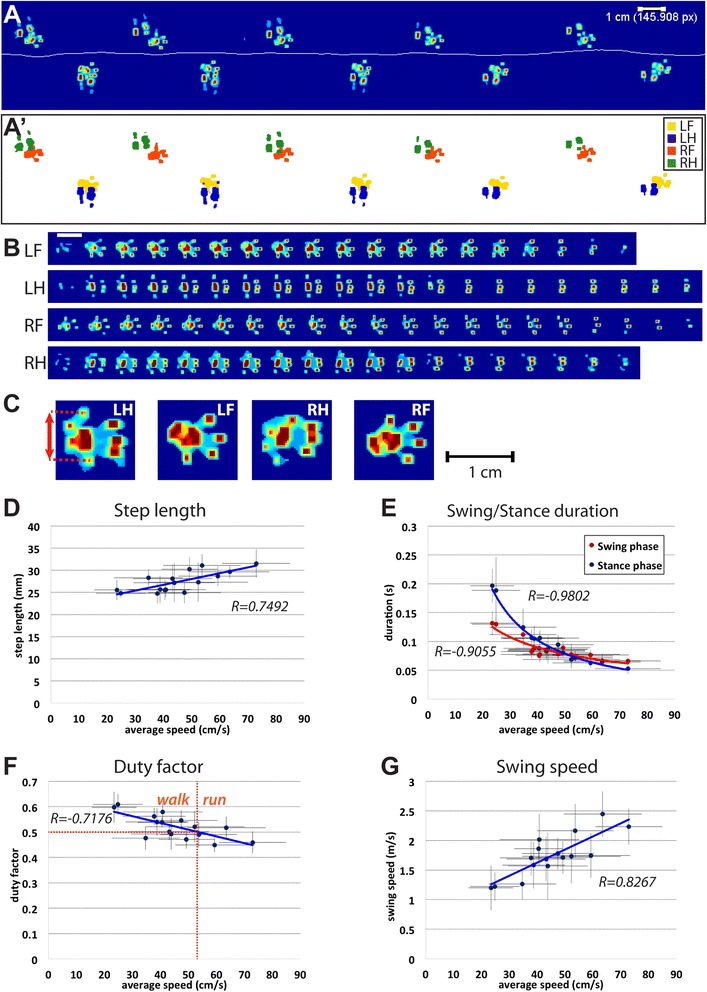
Footprint analysis tools and step parameters. **a** Complete footprint pattern. A heat map represents pixel intensity and the *horizontal line* represents the body path. **a'** Individual feet are labeled with different colors: the left fore, left hind, right fore, and right hind legs are represented in yellow, blue, orange, and green, respectively. **b** Stance phase dynamics. Each row shows successive frames for a single stance phase. All four legs are represented, *top* to *bottom*: left fore (LF), left hind (LH), right fore (RF), and right hind (RH). Stance initiation is to the left and each frame is 4 ms apart. White bar, 1 cm scale. **c** Individual full stance footprint. Each footprint is individually represented by a pixel intensity heat map. A pixel to centimeter conversion allows the user to measure changes in toe spreading for each footprint (*red arrows*). **d–g** Step parameters as a function of speed. Graphical fits are included. *x*-axis error bars represent standard deviations of the average speed. **d** Step length increases with speed. **e** Swing and stance phase durations are inversely proportional to speed. **f** Duty factor decreases with speed. Linear regression line (*y* = −0.0027*x* – 0.6425) determines that for speeds faster then 52.8 cm/s (*vertical dashed red line*), the duty factor falls below 0.5 (*horizontal dashed red line*), which confines the transition from a walk to a run-like gait. **g** Swing speed increases linearly with speed

The footprint test is a common qualitative readout that displays the footprint pattern and footprint contacts, potentially highlighting gait abnormalities [13]. MouseWalker can generate such a readout displaying the footprint pattern generated by the walking animal in addition to the path created by the body center (Fig. 3a). Footprints are converted to heat maps, as determined by the intensities of the fTIR signal, which are proportional to the pressure applied. Using a proper calibration procedure where, for example, all four paws are in contact with the acrylic surface while the animal is immobile, the user can quantify the pressure. Our data set displays the typical wild-type pattern with evenly spaced steps. Hind paw placement partially overlaps the previous forepaw placement, although offset at slightly lateral positions. Since this footprint overlap makes leg identification more challenging, an additional footprint pattern is generated where pixel intensity is eliminated by a leg-specific color code, allowing the unambiguous identification of the footprints from each leg (Fig. 3a' and Additional file 11: Video S5).

Because video acquisition is carried out at a high temporal resolution of 250 Hz, a series of frames spanning an entire stance phase can be generated. The data extracted from this series are referred to as *footprint dynamics*. At 250 Hz, we observed that the initial contact with the surface at touchdown is typically done with the entire paw touching the surface almost simultaneously, with most of the pressure exerted by the metatarsal and metacarpal pads on the hind and fore legs, respectively (Fig. 3b, left section). This characteristic is distinct from human walking, where stance phases begin with contact by the heel [25], or the cat, where contact is initiated by the most rostral section of the paw [26, 27]. During the stance phase in the mouse, pressure is gradually transferred rostrally to the toes prior to liftoff (Fig. 3b right section). The time spent when the stance phase was supported by the toes can be up to one-third of the entire stance phase (Fig. 3b). Visual inspection of the complete data set suggests that this behavior is independent of the walking speed (data not shown).

The MouseWalker program also compiles the complete set of footprints present in each video (Fig. 3c). We observed that at the end of the swing phase, just prior to touchdown, the toes changed their conformation from a closed to an actively open conformation (data not shown). This behavior occurs typically 20–30 ms before contact with the ground, indicating that it is under active neuronal control. Consistent with this notion, toe spreading has been used as a metric to study sciatic nerve function (for example [28, 29]). Due to the orientation of the footprints relative to the displacement axis, automatic quantification of toe spreading can be prone to errors. Nevertheless, each set of images generated by MouseWalker is provided with a scale calibration, allowing the user to measure distances easily and accurately using ImageJ from the National Institutes of Health (NIH) or similar software.

We also quantified several gait parameters as a function of walking speed (Fig. 3d–g). We observed that faster animals display ~20 % longer step strides, changing from ~25 mm in slower animals to ~30 mm in faster animals (Fig. 3d). A much larger variation was observed in stance phase duration (Fig. 3e), with values becoming exponentially shorter as speed increases, with a minimum of approximately 50 ms for the fastest animals. Swing duration exhibits a much smaller variation as observed in other experimental conditions [30, 31]. Although a similar variation is observed in invertebrate systems, stance phases last longer than swing phases at all speeds [21, 32]. In contrast, in our data set, we observed that above ~50 cm/s, swing duration is longer than stance phase duration, consistent with recent results [33]. We also used these data to measure the *duty factor*, defined as the fraction of the step cycle where the leg is in the stance phase (stance duration / period) [34]. This parameter has been used to distinguish walks from runs, as values ≥0.5 are described as walks, while values below 0.5 are considered runs [35, 36]. From our data, we find that swing duration surpasses stance duration at a duty factor of 0.5, which corresponds to a speed of 52.8 cm/s (Fig. 3f). Above this speed, the feet spend on average more time in the swing phase than in the stance phase, which is typical of running [34]. Accordingly, swing speeds also increase with increased speed (Fig. 3g).

**Fig. 4:**
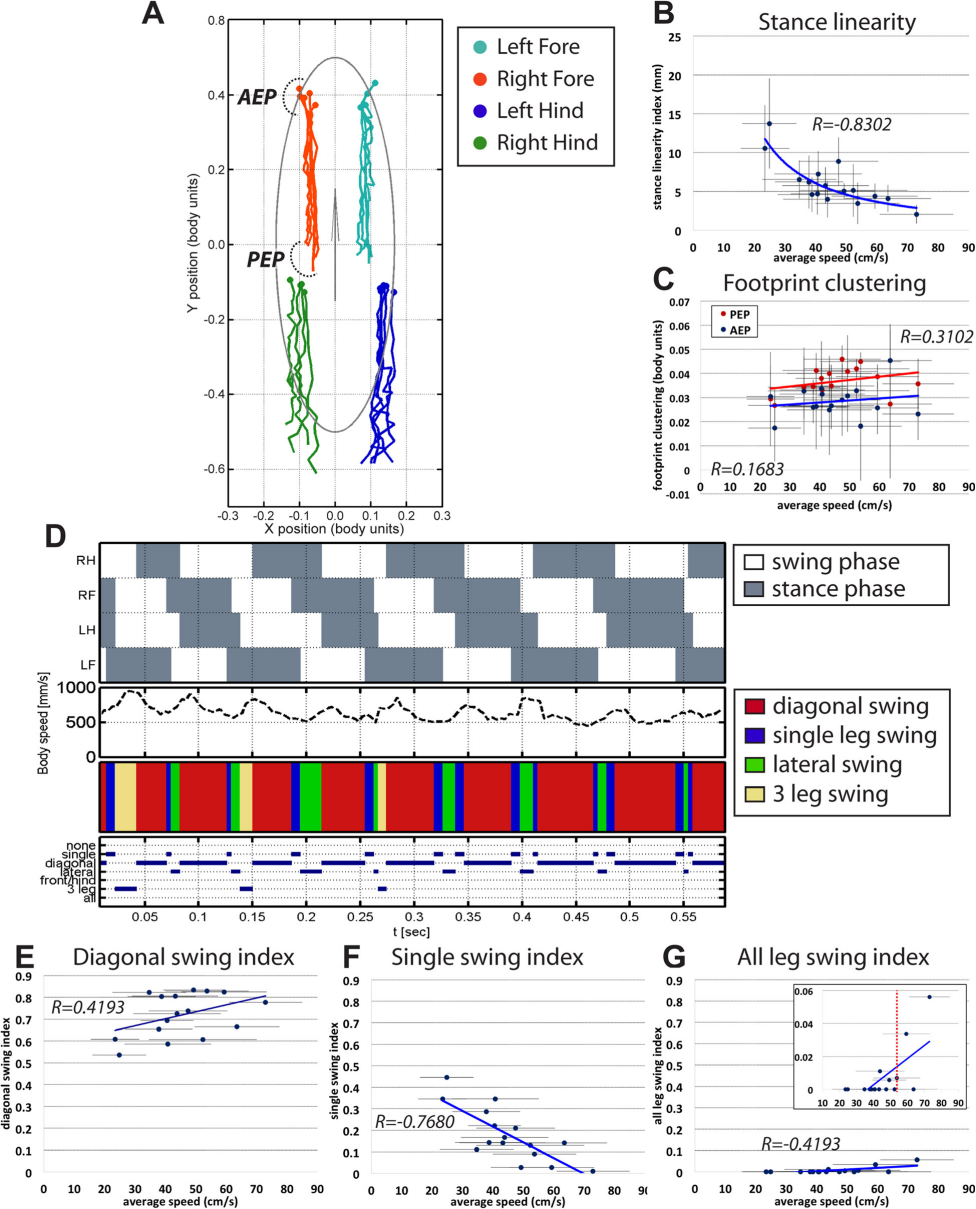
Stance traces and inter-leg coordination parameters. **a** Stance traces. Representative plot of an animal walking at 34.8 cm/s. Traces are generated by the position of the stance phase footprints relative to the body center (set at 0.0, 0.0). For each leg, stance onset corresponds to the anterior extreme position (AEP, marked by a *dashed line*) while stance offset is termed the posterior extreme position (PEP). **b** Stance linearity index decreases as a function of speed. Each data point measures the average jitter of the stance traces for all the legs. *x*-axis error bars represent standard deviations of the average speed. **c** Footprint clustering values are on average higher for PEP compared to AEP and increase with speed. **d** Gait patterns and step combinations. From *top* to *bottom*: Gait patterns (*white areas* represent swing phases and *gray areas* represent stance phases), instantaneous speed, color-coded leg conformation, and leg combination traces. **e–g** Leg combination indexes. *y*-axis upper limits are set to 0.9 to facilitate comparison. Graphical fits are also represented. **e** Diagonal swing index as a function of speed. Diagonal swing is the most representative leg combination, which increases with speed. **f** Single swing index decreases significantly with increased speed. **g** The all-legs swing index is observed primarily at higher speeds. The *inset* is the adjusted *y*-axis. The *vertical dashed line* is the transition from a walk to run-like behavior

Our MouseWalker software tracks not only footprints but also body features. Despite some tracking inaccuracies of the body center and footprint center, these measurements allow the stance phase of each leg to be reconstructed as it is anchored at the floor relative to the body (Fig. 4a and Additional file 12: Video S6). Thus, each stance trace reflects the amount of body wobble during stance phases. Each stance trace is normalized to body length to account for variations in body size and is defined as the position of the foot relative to the center of the body from paw touchdown (anterior extreme position, AEP) to the end of the stance phase (posterior extreme position, PEP) [21]. Regardless of the speed, stance traces run parallel to the body axis with the forelegs positioned more medially (Fig. 4a). A measure of the straightness of the stance traces, the *stance linearity index*, is calculated by computing the average difference between the actual stance trace and a smoothed version of the trace [21]. Similarly to what was observed for the fruit fly *Drosophila melanogaster*, stance traces become straighter as speed increases (Fig. 4b). In addition, we also measured the variability in the AEP and PEP coordinates for all steps in each video (Fig. 4c). This parameter, termed *footprint clustering*, corresponds to the standard deviation of the average AEP and PEP coordinates for each video [21]. Thus a smaller value for this parameter corresponds to a more consistent position for paw touchdown or takeoff (AEP or PEP, respectively). As with *Drosophila*, we observed smaller footprint clustering values for AEP compared to PEP, indicating a more consistent foot placement at the onset of the stance phase, possibly due to tighter motor control. In contrast to the fly, where faster animals had smaller footprint clustering values, there was little dependence on speed for this parameter in the mouse [21].

Adult mice are typically described as using a *walk* gait at slower speeds, a *trot* gait at intermediate speeds, and a *gallop* or *bound* gait at higher speeds [33, 37], although additional variants have been described [36, 38]. The walk gait is generally defined as when only one leg is swinging, a trot is defined by the simultaneous swinging of diagonal feet, and a gallop has two defining characteristics: only a single foot is in the stance phase and there is an aerial phase (no feet are in the stance phase) [39]. With these definitions in mind, MouseWalker outputs the step pattern with the instantaneous speed and step combinations associated with each frame in the video (Fig. 4d). Although videos are selected based on the animal’s average speed, plots of instantaneous speed display a wave-like appearance with minimum speeds typically occurring at step transitions (Fig. 4d). These observations are consistent with other experimental conditions in mice as with other quadrupeds and walking insects [21, 31, 40, 41]. As a proxy for assessing the presence of specific gaits, MouseWalker computes the fraction of frames assigned to a particular leg combination (Fig. 4e–g and Additional file 13: Figure S6). We defined seven categories for the possible stance combinations: *no swing*, *single-leg swing* (regardless of the position), *diagonal-leg swing*, *lateral-leg swing* (both left or both right legs), *front or hind swing* (both hind or both fore legs), *three-leg swing*, or *all-legs swing*. In our data set, the two diagonal swing conformations, which are typically observed in the trot gait, are the most representative configuration, present in more than 50 % of the frames (Fig. 4e). The presence of this gait pattern increases with speed, reaching approximately 80 % even at intermediate speeds. Concurrently, there is a decrease in the fraction of frames in which only a single leg is swinging (Fig. 4f), typical of walk gaits, and in the fraction of frames with all feet in the stance phase (Additional file 13: Figure S6A). We also observed an increase in the fraction of frames with three legs in a swing position, typical of gallop gaits (Additional file 13: Fig. S6B). These results reveal that there is progressively reduced contact with the ground as speed increases. Consistently, for speeds greater than 52.8 cm/s, the mice have a duty factor below 0.5 (Fig. 3f), suggesting the presence of an aerial phase [34]. We find that for most of the animals with a duty factor below 0.5, and thus are considered to show a run-like behavior, there are some frames in which all legs are in the swing phase (Additional file 14: Table S2 and Fig. 4g). The MouseWalker program also calculates the fraction of lateral swing conformations, typical of the *pace* gait (Additional file 13: Figure S6C), and simultaneous front or hind swing, found in *bound* and *hopping* gaits (Additional file 13: Figure S6D). In our data set, we found a poor correlation between these step configurations and speed (Additional file 13: Figure S6C, D). Although most laboratory strains rarely display bound or hopping gaits [33], some mouse species and genetically modified mice that lack left–right coordination use these gaits more frequently [38, 42].

Finally, the MouseWalker software package also analyzes phase values between contralateral legs (Additional file 13: Figure S6E), which provide a measure of inter-leg coordination. As expected, these measurements indicate that contralateral legs move in anti-phase with phase values of 0.507 ± 0.041 and 0.491 ± 0.061 for fore and hind legs, respectively.

## Discussion

The quantification of rodent motor patterns is important for the phenotypic characterization of motor outputs in a wide variety of experimental conditions, including human disease models, gene and circuit disruption studies, following induced brain or spinal cord injury, and in response to drugs. Motor assays should provide a thorough, cost-effective and minimally laborious approach to describing shifts in locomotor performance compared to a baseline or control group.

Here we describe a simple and easily accessible fTIR arena combined with a complete and open-source software package to track and quantify the walking behavior of rodents without the use of any body markers. Using this approach, we measured a large number of parameters and readouts, which provide researchers with an easily accessible method for describing locomotor defects quantitatively and qualitatively. Many of the described outputs, such as footprint patterns or toe-opening measurements, are typical metrics of motor function (Fig. 3a, c), while other parameters, such as stance linearity or footprint clustering, have not been described previously for rodents (Fig. 4b, c). Importantly, these parameters have been shown to provide a sensitive readout for motor coordination in the fruit fly [21, 22]. Our freely available software package does not require expertise in programing, as it can be used as a self-contained executable file format or as a MATLAB script. The former mode also allows the user to modify the existing script to extract additional kinematic parameters or change the output files. Importantly, the software can interpret both black and white and color videos, which increases the range and functions of compatible video cameras. Moreover, the fTIR arena can be easily redesigned with larger walking surfaces to accommodate larger animals or groups of animals. This setup can also be combined with other kinematic and physiological readouts, such as stick diagrams or electromyographic recordings.

Both versions of the software, the documentation in addition to detailed instructions for building and using the fTIR apparatus can be downloaded for free [43]. We expect that this approach will stimulate the inclusion of highly quantitative kinematic features for phenotypic characterization.

## Conclusion

Here we present a simple and readily applied approach to quantitatively describe rodent locomotion with high precision and detail. Our user-friendly software package provides a large set of parameters covering multiples aspects of locomotion, which will facilitate comparison between control and experimental groups. In addition, the affordable and easy to build setup will allow a larger number of researchers to study motor behavior under wild type or aberrant conditions and unambiguously measure relevant phenotypes.

## Methods

### MouseWalker apparatus

The MouseWalker apparatus primarily comprises four components: the fTIR floor and walkway wall, the supporting posts, the 45° mirror, and the background light (see Additional file 1: Figure S1, Additional file 2: Figure S2, Additional file 3: Figure S3, Additional file 4: Figure S4, Additional file 5: Figure S5 for additional details).

#### The fTIR floor and walkway wall

A cool white LED light strip for black and white cameras or a colored LED light strip for color cameras (HitLights, LA, USA, parts # SDM3528-120LED/M or HL-LS5050_RGB300NW44K, respectively) was glued to a 3/8-inch U-channel aluminum base LED mount (MacMaster-Carr, IL, USA, part # 9001 K12). This LED/aluminum bar was clamped to the long edges of a 9.4-mm (3/8-inch) thick piece of acrylic glass measuring 8 by 80 cm (Additional file 2: Figure S2). A strip of black cardboard was glued and sewn over the LED/acrylic glass contact areas. To build the acrylic glass walkway, all four sides were glued together with epoxy glue and cable ties (see Additional file 3: Figure S3 for dimensions and details) and placed over the fTIR floor.

#### Supporting posts

The supporting posts were made by gluing a 1-inch and a 1/2-inch hollow acrylic glass base, each 12 inch tall, to a 2-inch-square base. Approximately at the middle section of a post, two 3/4-inch (19 mm) U-channel aluminum pieces (MacMaster-Carr, IL, USA, part # 9001 K25), 9 and 13 cm long, were each clamped by two 10–32 button head 2-inch-long screws. The fTIR floor sits over the 13-cm aluminum base section and is clamped by rubber bands (see Additional file 2: Figure S2 for details). Two spring clamps secured the walkway wall, which sits on top of the fTIR floor, to the hollow supporting posts.

#### 45° mirror

A 1/4-inch-thick mirror (Dulles Glass and Mirror, VA, USA) measuring 77.94 × 22.54 cm (30 11/16 × 8 7/8 inch), was mounted on four laser-cut acrylic glass bases and connected by three stud screws (see Additional file 4: Figure S4 for details).

#### Background backlight

A colored LED light strip (80 cm long; HitLights, LA, USA, part # HL-LS5050_RGB300NW44K) was glued to the inside face of a 3/4-inch U-channel aluminum bar (80 cm long; MacMaster-Carr, IL, USA, part # 9001 K25). Two LED/aluminum bar sets were assembled at the longer edge of an 80 × 15 cm 3/8-inch-thick transparent piece of acrylic glass covered on one face by white opaque adhesive vinyl and on the opposing face by semitransparent adhesive vinyl. Each LED/aluminum bar was further clamped to the acrylic glass by three M6 screws distributed along the aluminum bar. The color was set by a controller box and remote control. The backlight board was placed 40 cm over the fTIR apparatus and supported by a stand (see Additional file 5: Figure S5 for details).

### Video acquisition

Four C57BL/6J strain female mice weighing between 19.8 and 22.3 g were used. All animal protocols followed NIH guidelines and were approved by Columbia University’s Institutional Animal Care and Use Committee under the protocol AC-AAAD8755. Four videos were quantified for each animal. Each data point is generated by one video.

Movies were acquired using a Gazelle 2.2-MP camera (Point Grey, Richmond, Canada) mounted on a tripod and connected to a Makro-Planar T 2/50 lens (Carl Zeiss, Jena, Germany) at maximum aperture (f/2.0) to increase light sensitivity and minimize depth of field. Video recordings were controlled and exported using StreamPix6 software (NorPix, Montreal, Canada) and subsequently cropped using an ImageJ macro (Christian Liebig, MPI for Developmental Biology, Germany).

### MouseWalker program

The MouseWalker program was developed and compiled in MATLAB (The Mathworks, MA, USA). Both the program and manual are available online [43]. The body and footprints of the mouse are distinguished from the background and from each other based on their color or pixel intensity. The background is identified separately for each pixel in the images, around the median color when the mouse is not present in the given pixel, within a red/green/blue (RGB) range that accounts for background brightness fluctuations. The RGB color of the mouse body and footprints can be defined by the user based on the setup. Within the body of the mouse, the tail is identified as a consecutive part of the body below a thickness threshold, starting from the posterior end of the tail. Three equidistant points along the tail are stored, with their orientation within the tail, which can be used to characterize tail curvature. Within the rest of the body, the end of the nose is identified. From the nose, the head is defined as the part of the body within a distance threshold from the nose. The center and direction of this head part are also stored. The center of the body without the tail and its orientation are stored, the latter being the orientation along the major axis of the body without the tail. A body "back" point is also identified and stored along with its direction, which is the point halfway between the body center and the start of the tail. For the footprints of the animal, the number of pixels within a footprint, as well as the sum of the brightness of these pixels, are stored by the software.

### Parameters quantified by MouseWalker

- Speed: instantaneous and average (cm/s)
- Frequency (cycles/s)
- Period (ms)
- Swing speed: average and for individual steps (m/s)
- Step length: average and for individual steps (mm)
- Swing time: average and for individual steps (s)
- Stance time: average and for individual steps (s)
- Duty factor (unitless)
- AEP (body units)
- PEP (body units)
- Footprint clustering (AEP and PEP; body units)
- Stance linearity index: average and for individual segments (m)
- Body linearity index (mm)
- Leg combination indexes: no swing, single-leg swing, diagonal-leg swing, lateral-leg swing, front or hind swing, three-leg swing, or all-legs swing (unitless)
- Leg phases: front and hind legs (unitless)
- Footprint pixel intensity (unitless)
- Footprint area (cm^2^)

### List of files generated by MouseWalker

- Angle between footprint and displacement axis vs. time
- Footprint distance to body center vs. time
- Footprint parallel distance to body center vs. time
- Footprint perpendicular distance to body center vs. time
- Instantaneous speed vs. time
- Gait vs. time
- Combined gait pattern, instantaneous speed, color-coded gait pattern, and leg combination traces over time (fixed and automatic timescale)
- AEP plus stance trace
- Summary plots including combined gait pattern over time, instantaneous speed, leg combination traces, and footprint area, plus a stance trace
- Excel file with all parameters and tracking data
- Table of footprints
- Image sequence from the tracking program
- Orientation of body elements compared to the orientation of the body as a whole
- Proximal and distal tail orientation compared to the orientation of the middle of the tail
- Tail perpendicular velocity over time
- Tail orientation vs. body axis over time
- Foot dynamics for all stance phases for all feet
- Footprint pattern, pixel intensity
- Footprint pattern, color coded (fixed and automatic timescale)
- Leg combination traces (fixed and automatic timescale)
- Leg combination color code
- Footprint area over time
- Footprint pixel intensity over time
- Footprint pixel intensity/area (pressure) over time

### Availability of data and materials

The MouseWalker software is freely available online [43] for Windows XP and above. The software was written in MATLAB.

## Additional files

Additional file 1: Figure S1. fTIR apparatus and individual components. Additional file 2: Figure S2. fTIR floor and support base. Individual components are also listed. Additional file 3: Figure S3. Walkway wall. Dimensions of individual parts are also listed. Additional file 4: Figure S4. 45° mirror base. Individual components are also listed. Additional file 5: Figure S5. Background light box. Individual components are also listed. Additional file 6: Video S1. fTIR effect. Additional file 7: Video S2. Tracked fTIR video. Additional file 8: Video S3. Tracked fTIR video, color coded. Additional file 9: Video S4. Tracked fTIR video, just footprints. Additional file 10: Table S1. Parameters on the Excel file, sheet 1. Additional file 11: Video S5. Tracked fTIR video, footprints color-coded. Additional file 12: Video S6. Tracked fTIR video, body locked. Additional file 13: Figure S6. Leg combination indexes and leg phases. (A-D) Graphical fits are included. x-axis error bars represent standard deviations of the average speed. y-axis upper limits are set to 0.9 to ease comparison with Fig. 4E-G. Graphical fits are also represented. (A) No swing index. (B) Three leg swing index. (C) Lateral swing index. (D) Front/hind swing index (E) Circular plots of contralateral leg phases walking animals. Blue dots indicate individual phase values for all 16 videos. N=54 and 49 for front and hind legs, respectively. Mean vectors represented a red arrow. Inner red circle indicates a Rayleigh p value of 0.05. Additional file 14: Table S2. Videos of walking mice with a duty factor higher then 0.5 correspond to a walk-like behavior are highlighted in green. Videos with a duty factor higher then 0.5 correspond to a run-like behavior are highlighted in red. With the exception of one recording, all running animals display frames with an areal phase (underscored).

## Abbreviations

AEP: anterior extreme position
fTIR: frustrated total internal reflection
NIH: National Institutes of Health
PEP: posterior extreme position

## References

[1] Goulding M (2009). Circuits controlling vertebrate locomotion: moving in a new direction. Nat Rev Neurosci..

[2] Kiehn O, Dougherty KJ, Hagglund M, Borgius L, Talpalar A, Restrepo CE (2010). Probing spinal circuits controlling walking in mammals. Biochem Biophys Res Commun..

[3] Wong PC, Cai H, Borchelt DR, Price DL (2002). Genetically engineered mouse models of neurodegenerative diseases. Nat Neurosci..

[4] Drai D, Kafkafi N, Benjamini Y, Elmer G, Golani I (2001). Rats and mice share common ethologically relevant parameters of exploratory behavior. Behav Brain Res..

[5] Wallace JE, Krauter EE, Campbell BA (1980). Motor and reflexive behavior in the aging rat. J Gerontol..

[6] Jones BJ, Roberts DJ (1968). The quantitative measurement of motor inco-ordination in naive mice using an accelerating rotarod. J Pharm Pharmacol..

[7] Azim E, Jiang J, Alstermark B, Jessell TM (2014). Skilled reaching relies on a V2a propriospinal internal copy circuit. Nature..

[8] Hickey MA, Kosmalska A, Enayati J, Cohen R, Zeitlin S, Levine MS, Chesselet MF (2008). Extensive early motor and non-motor behavioral deficits are followed by striatal neuronal loss in knock-in Huntington's disease mice. Neuroscience.

[9] Leblond H, L'Esperance M, Orsal D, Rossignol S (2003). Treadmill locomotion in the intact and spinal mouse. J Neurosci..

[10] Pearson KG, Acharya H, Fouad K (2005). A new electrode configuration for recording electromyographic activity in behaving mice. J Neurosci Methods..

[11] Tysseling VM, Janes L, Imhoff R, Quinlan KA, Lookabaugh B, Ramalingam S (2013). Design and evaluation of a chronic EMG multichannel detection system for long-term recordings of hindlimb muscles in behaving mice. J Electromyogr Kinesiol..

[12] Akay T, Tourtellotte WG, Arber S, Jessell TM (2014). Degradation of mouse locomotor pattern in the absence of proprioceptive sensory feedback. Proc Natl Acad Sci USA.

[13] Carter RJ, Lione LA, Humby T, Mangiarini L, Mahal A, Bates GP (1999). Characterization of progressive motor deficits in mice transgenic for the human Huntington's disease mutation. J Neurosci..

[14] Hamers FP, Koopmans GC, Joosten EA (2006). CatWalk-assisted gait analysis in the assessment of spinal cord injury. J Neurotrauma..

[15] Hamers FP, Lankhorst AJ, van Laar TJ, Veldhuis WB, Gispen WH (2001). Automated quantitative gait analysis during overground locomotion in the rat: its application to spinal cord contusion and transection injuries. J Neurotrauma..

[16] CatWalk, Noldus Information Technology, The Netherlands. http://www.noldus.com/animal-behavior-research/products/catwalk.

[17] RunwayScan, Clever Sys Inc., VA, USA. http://cleversysinc.com/csi_products/runwayscan.

[18] Pratte M, Jamon M (2009). Detection of social approach in inbred mice. Behav Brain Res..

[19] de Chaumont F, Coura RD, Serreau P, Cressant A, Chabout J, Granon S (2012). Computerized video analysis of social interactions in mice. Nat Methods..

[20] Berman GJ, Choi DM, Bialek W, Shaevitz JW. Mapping the stereotyped behaviour of freely moving fruit flies. J R Soc Interface. 2014;11:20140672. http://www.ncbi.nlm.nih.gov/pmc/articles/PMC4233753/. 10.1098/rsif.2014.0672 PMC4233753 25142523

[21] Mendes CS, Bartos I, Akay T, Marka S, Mann RS (2013). Quantification of gait parameters in freely walking wild type and sensory deprived *Drosophila melanogaster*. Elife..

[22] Mendes CS, Rajendren SV, Bartos I, Marka S, Mann RS (2014). Kinematic responses to changes in walking orientation and gravitational load in *Drosophila melanogaster*. PLoS One..

[23] Betts RP, Duckworth T (1978). A device for measuring plantar pressures under the sole of the foot. Eng Med..

[24] Sumriddetchkajorn S, Amarit R (2006). Ultra-high-contrast low-leakage-light optical touch device structures using light scattering and total internal reflection concepts. Sens Actuators A Phys..

[25] Lieberman DE, Venkadesan M, Werbel WA, Daoud AI, D'Andrea S, Davis IS (2010). Foot strike patterns and collision forces in habitually barefoot versus shod runners. Nature..

[26] Bouyer LJ, Rossignol S (2003). Contribution of cutaneous inputs from the hindpaw to the control of locomotion. I. Intact cats. J Neurophysiol..

[27] Bouyer LJ, Rossignol S (2003). Contribution of cutaneous inputs from the hindpaw to the control of locomotion. II. Spinal cats. J Neurophysiol..

[28] Elfar JC, Jacobson JA, Puzas JE, Rosier RN, Zuscik MJ (2008). Erythropoietin accelerates functional recovery after peripheral nerve injury. J Bone Joint Surg Am..

[29] Kato N, Matsumoto M, Kogawa M, Atkins GJ, Findlay DM, Fujikawa T (2013). Critical role of p38 MAPK for regeneration of the sciatic nerve following crush injury in vivo. J Neuroinflamm..

[30] Grillner S, Brooks VB (1981). Control of locomotion in bipeds, tetrapods and fish. Handbook of physiology: The nervous system, motor control Vol 2.

[31] Akay T, Acharya HJ, Fouad K, Pearson KG (2006). Behavioral and electromyographic characterization of mice lacking EphA4 receptors. J Neurophysiol..

[32] Wilson DM (1966). Insect walking. Annu Rev Entomol..

[33] Bellardita C, Kiehn O (2015). Phenotypic characterization of speed-associated gait changes in mice reveals modular organization of locomotor networks. Curr Biol..

[34] Hildebrand M (1965). Symmetrical gaits of horses. Science..

[35] McNeill AR (2002). Energetics and optimization of human walking and running: The 2000 Raymond Pearl memorial lecture. Am J Hum Biol..

[36] Biknevicius AR, Reilly SM (2006). Correlation of symmetrical gaits and whole body mechanics: debunking myths in locomotor biodynamics. J Exp Zool A Comp Exp Biol..

[37] Alexander RM (1982). Walking, running and jumping. Locomotion of animals.

[38] Eilam D (1997). Postnatal development of body architecture and gait in several rodent species. J Exp Biol..

[39] Biancardi CM, Minetti AE (2012). Biomechanical determinants of transverse and rotary gallop in cursorial mammals. J Exp Biol..

[40] Bertram JEA, Gutmann A (2009). Motions of the running horse and cheetah revisited: fundamental mechanics of the transverse and rotary gallop. J R Soc Interface..

[41] Graham D (1972). A behavioural analysis of the temporal organisation of walking movements in the 1st instar and adult stick insect (*Carausius morosus*). J Comp Physiol..

[42] Talpalar AE, Bouvier J, Borgius L, Fortin G, Pierani A, Kiehn O (2013). Dual-mode operation of neuronal networks involved in left-right alternation. Nature..

[43] MouseWalker. http://biooptics.markalab.org/MouseWalker/. doi:10.5281/zenodo.18233.

