# Supplementary figures and images for: Quantification of gait parameters in freely walking rodents
Source: BMC Biol. 2015 Jul 22;13:50. doi: 10.1186/s12915-015-0154-0 (PMC4511453; doi:10.1186/s12915-015-0154-0)

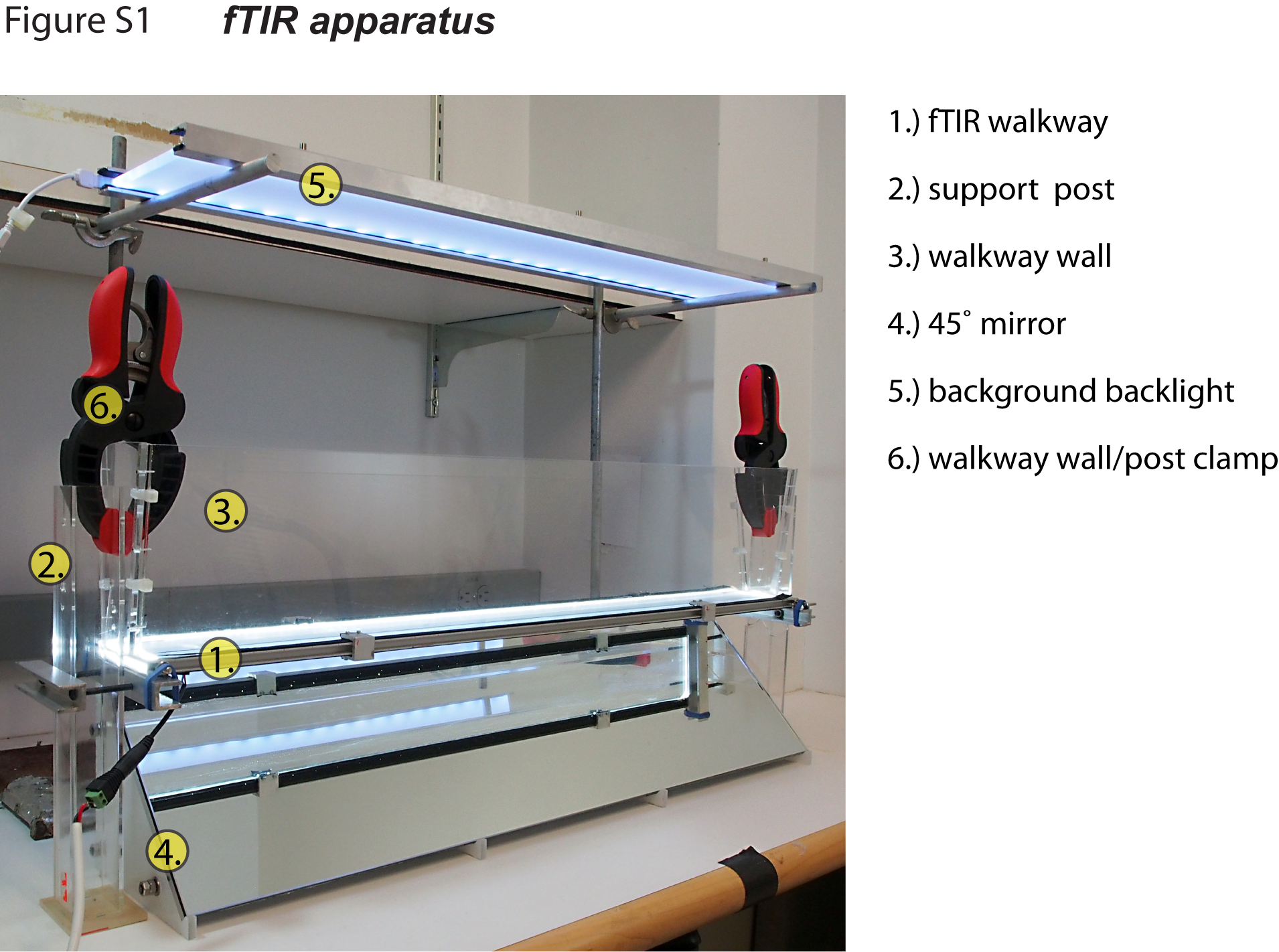

Supplement: Additional file 1: Figure S1. — fTIR apparatus and individual components. [file 12915_2015_154_MOESM1_ESM.tif]

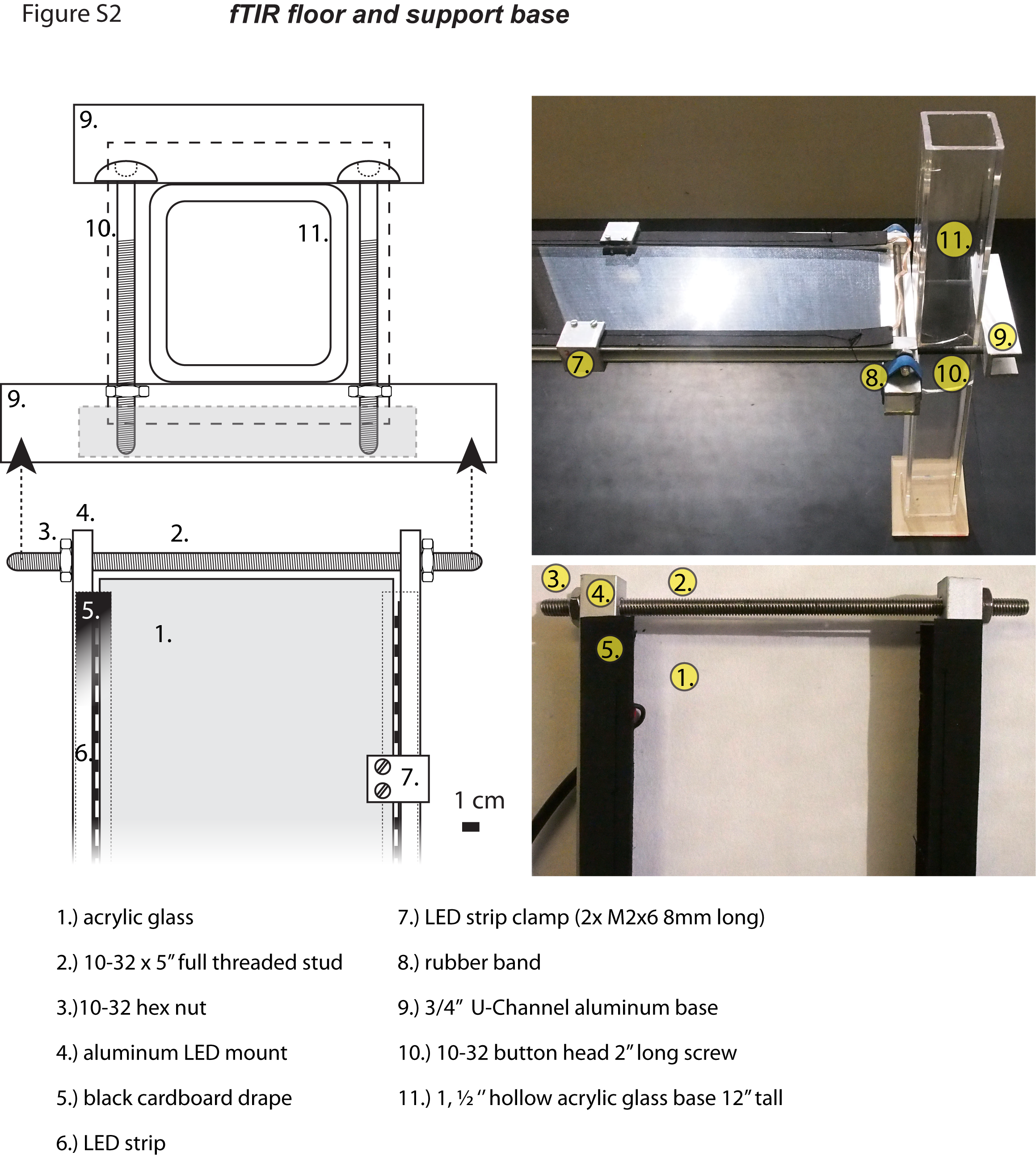

Supplement: Additional file 2: Figure S2. — fTIR floor and support base. Individual components are also listed. [file 12915_2015_154_MOESM2_ESM.tif]

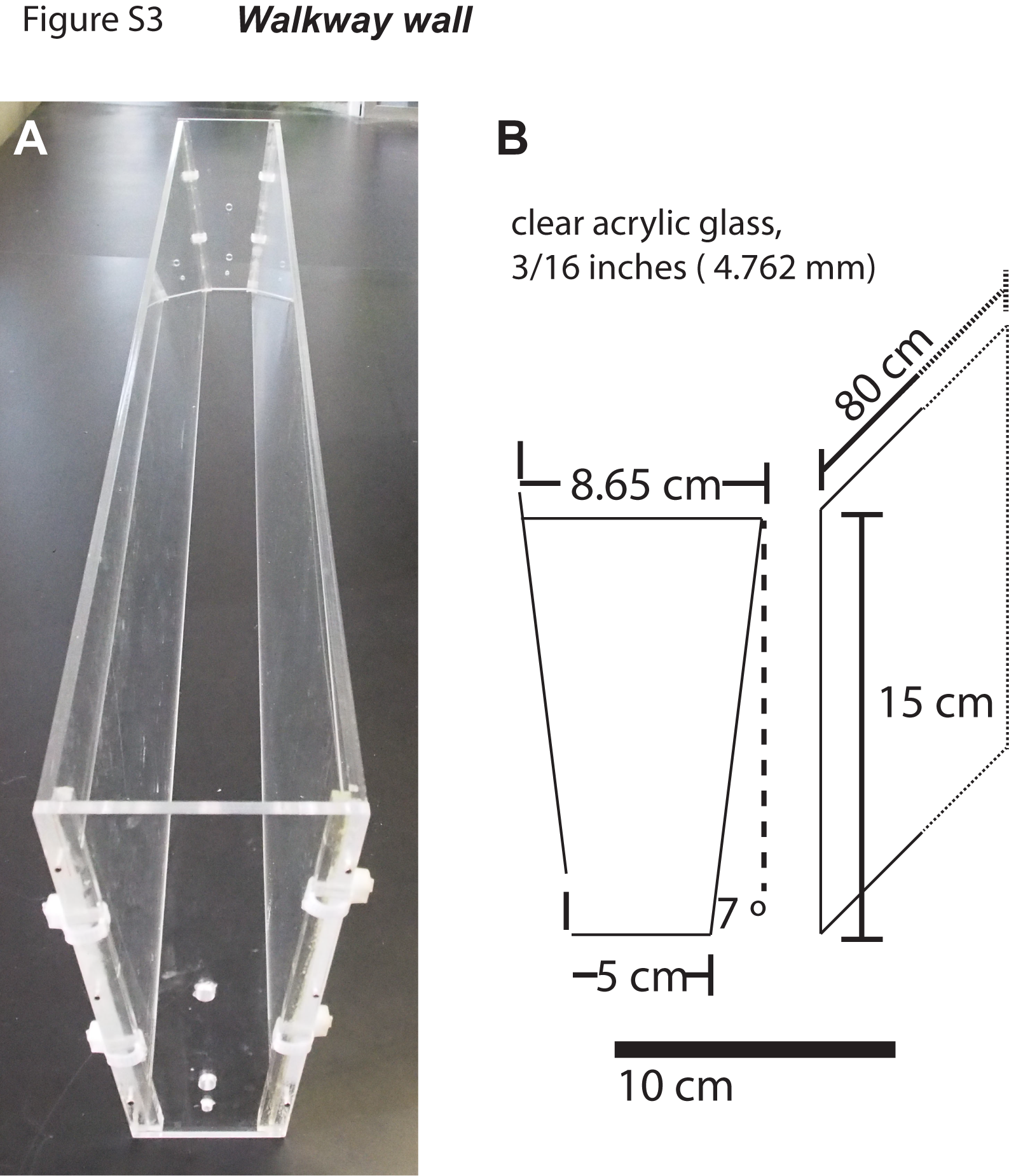

Supplement: Additional file 3: Figure S3. — Walkway wall. Dimensions of individual parts are also listed. [file 12915_2015_154_MOESM3_ESM.tif]

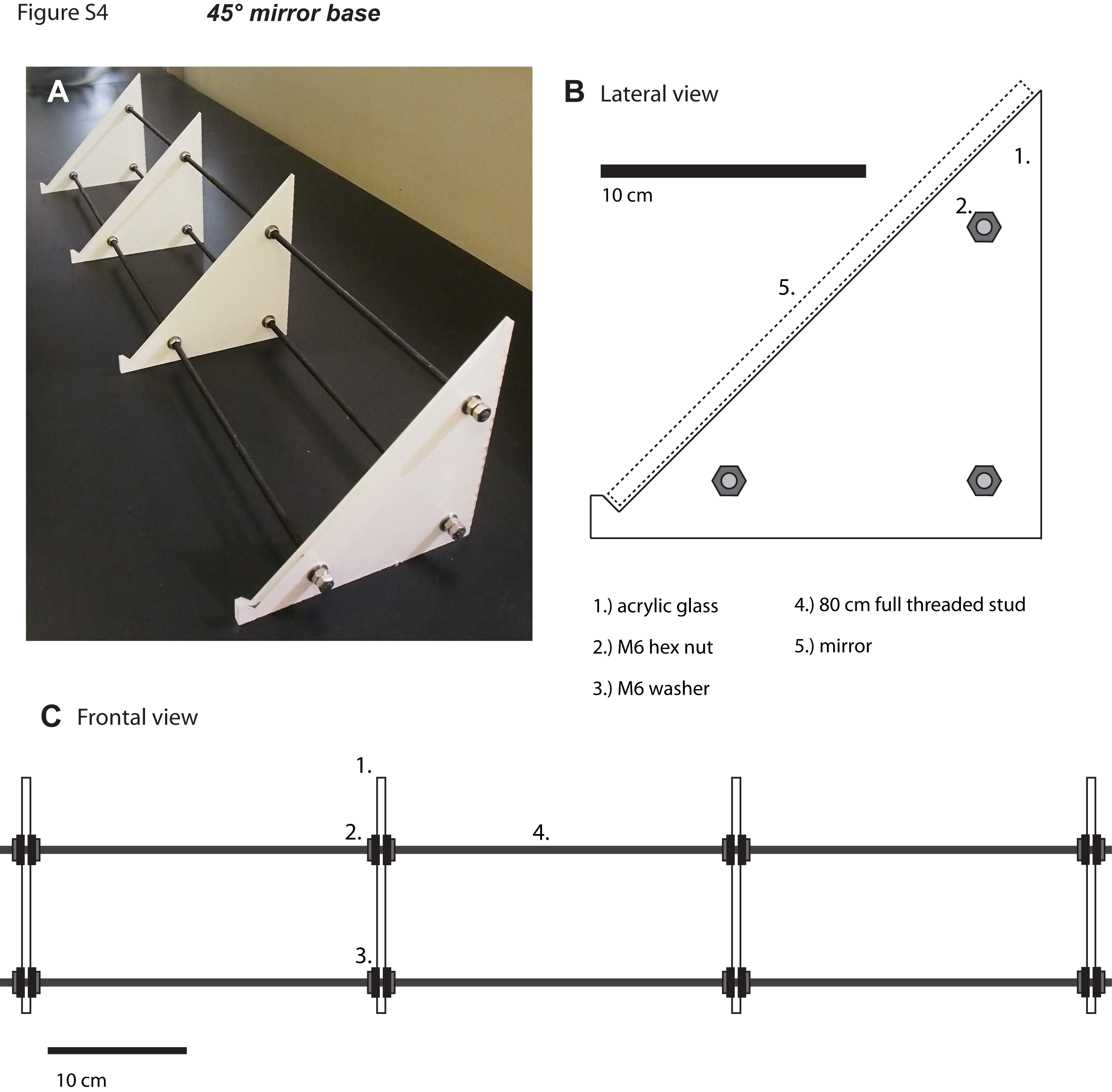

Supplement: Additional file 4: Figure S4. — 45° mirror base. Individual components are also listed. [file 12915_2015_154_MOESM4_ESM.tif]

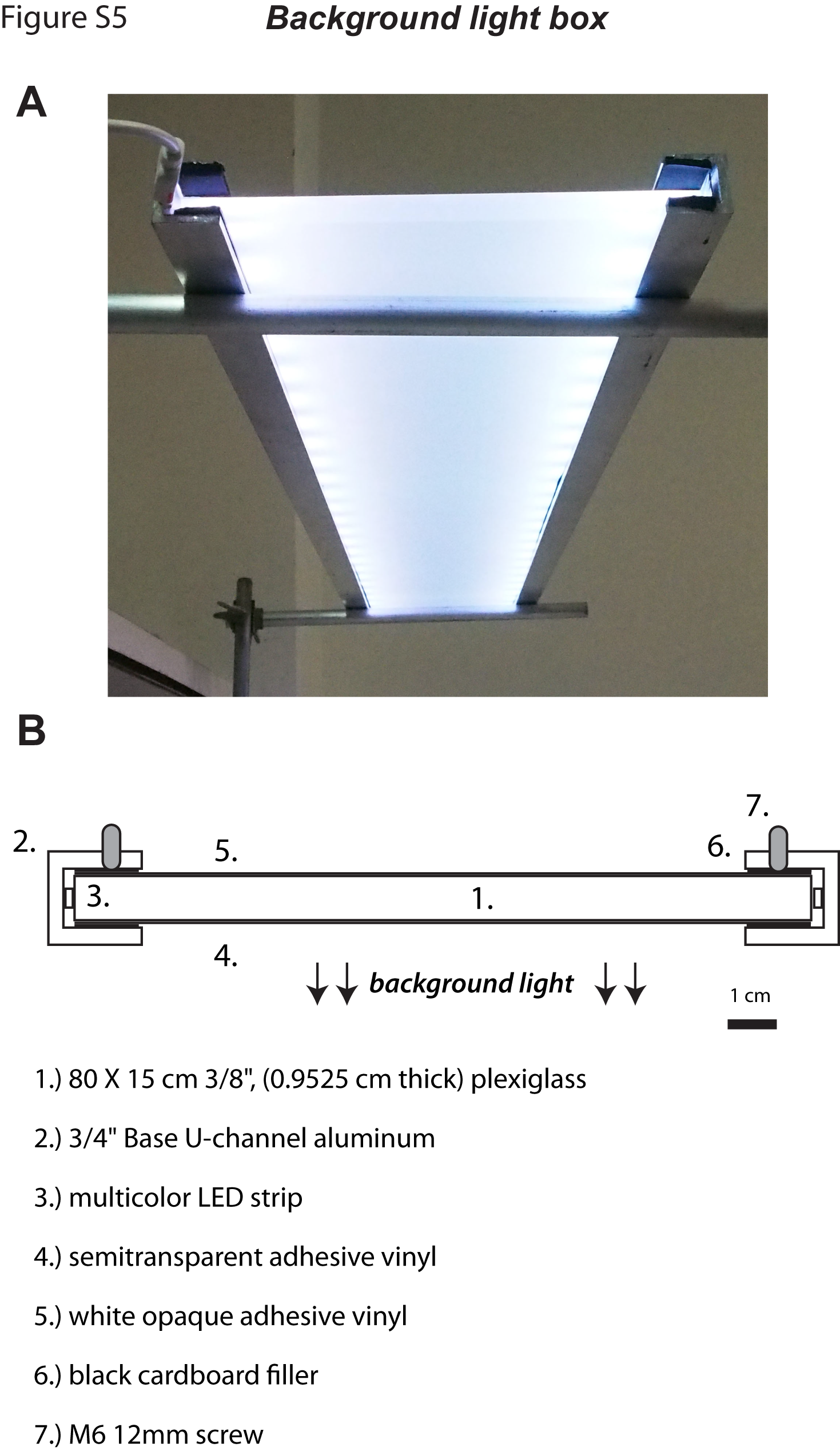

Supplement: Additional file 5: Figure S5. — Background light box. Individual components are also listed. [file 12915_2015_154_MOESM5_ESM.tif]

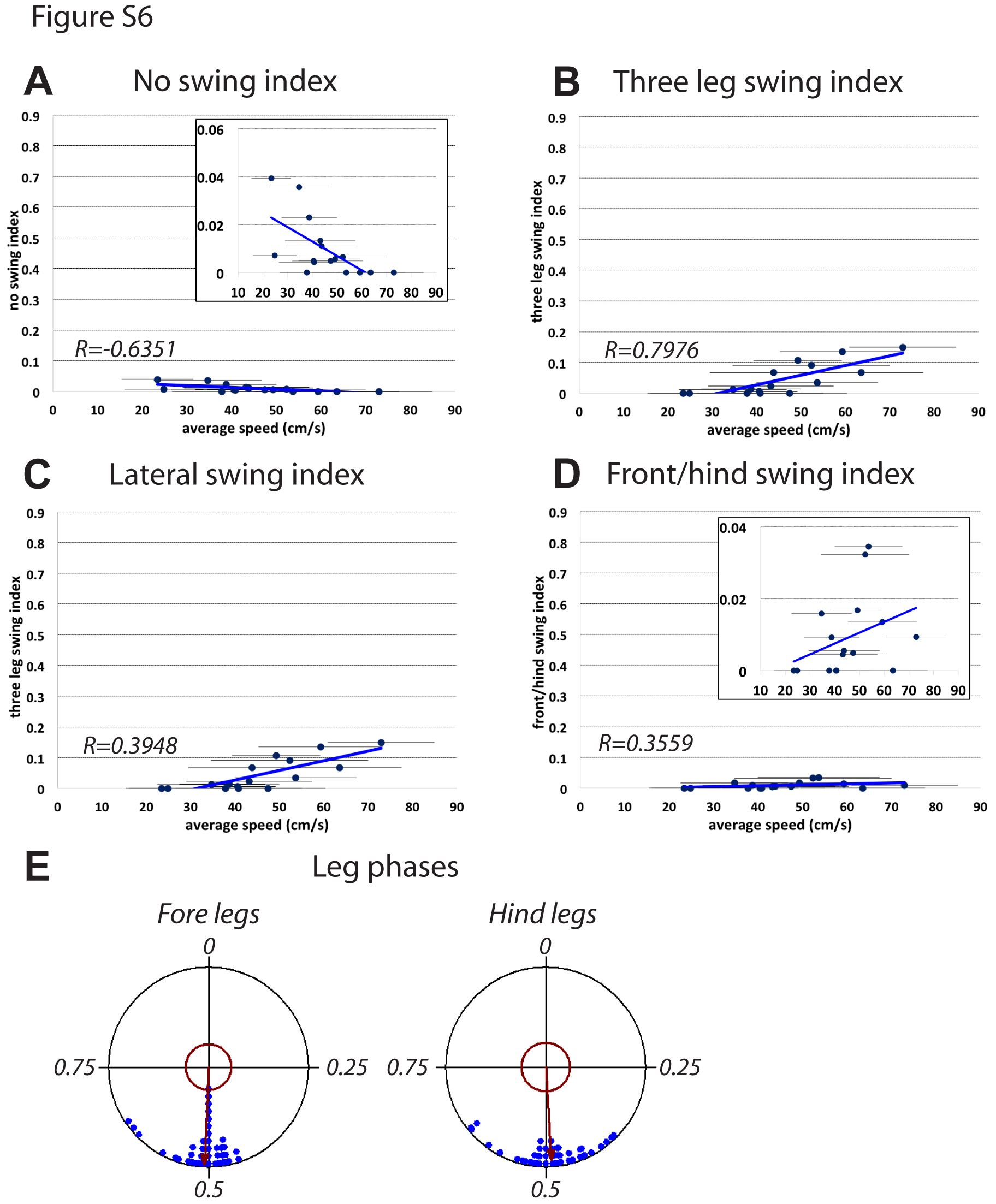

Supplement: Additional file 13: Figure S6. — Leg combination indexes and leg phases. (A-D) Graphical fits are included. x-axis error bars represent standard deviations of the average speed. y-axis upper limits are set to 0.9 to ease comparison with Fig. 4E-G. Graphical fits are also represented. (A) No swing index. (B) Three leg swing index. (C) Lateral swing index. (D) Front/hind swing index (E) Circular plots of contralateral leg phases walking animals. Blue dots indicate individual phase values for all 16 videos. N=54 and 49 for front and hind legs, respectively. Mean vectors represented a red arrow. Inner red circle indicates a Rayleigh p value of 0.05. [file 12915_2015_154_MOESM13_ESM.tif]
