# Supplementary material for: Quantification of gait parameters in freely walking rodents
Source: BMC Biol. 2015 Jul 22;13:50. doi: 10.1186/s12915-015-0154-0 (PMC4511453; doi:10.1186/s12915-015-0154-0)
Supplement: Additional file 10: Table S1. — Parameters on the Excel file, sheet 1. [file 12915_2015_154_MOESM10_ESM.docx]

**Supplemental Table 1**

*Parameters on sheet 1*

***Cell Parameter Description***

A label File name

B speed cm/s Average speed

C diag swing Diagonal swing index (Trot)

D single swing Single swing index (Walk)

E lateral swing Lateral swing index (Pace)

F freq Frequency, cycles / second

G period ms Period

H no swing No swing index (Stand)

I front/hind swing Front/hind swing index (Bound/hop)

J sw v(AVR) Average swing velocity for all legs

K sw v(SD) Standard deviation (STD) for swing velocity for all legs

L sw v(F) Average swing velocity for front legs

M sw v(H) Average swing velocity for hind legs

N sw s(AVR) Average step distance for all legs

O sw s(SD) Standard deviation for step distance for all legs

P sw s(F) Average step distance for front legs

Q sw s(H) Average step distance for hind legs

R sw t(AVR) Average swing time for all legs

S sw t(SD) Standard deviation for swing time for all legs

T sw t(F) Average swing time for front legs

U sw t(H) Average swing time for hind legs

V period SD Step period standard deviation

W stc t(F) Average stance time for front legs

X stc t(H) Average stance time for hind legs

Y stc t(AVR) Average stance time for all legs

Z stc t(SD) Standard deviation for stance time for all legs

AA varAEP_F Difference between the L/R front perpendicular AEP distance

AB varAEP_H Difference between the L/R hind perpendicular AEP distance

AC varPEP_F Difference between the L/R perpendicular PEP distance

AD varPEP_H Difference between the L/R perpendicular PEP distance

AE stnc stabl Averaged stance linearity for all legs.

AF SD AEP Average standard deviation for PEP

AG SD PEP Average standard deviation for AEP

AH F AEP Average distance of front AEP to the center of the body

AI H AEP Average distance of hind AEP to the center of the body

AJ F PEP Average distance of front PEP to the center of the body

AK H PEP Average distance of hind PEP to the center of the body

AL F AEPy Average position of front AEP to the center of the body, y axis

AM H AEPy Average distance of hind AEP to the center of the body, y axis

AN F PEPy Average distance of front PEP to the center of the body, y axis

AO H PEPy Average distance of hind PEP to the center of the body, y axis

AP F AEPySD Average position of front AEP to the center of the body, STD y axis

AQ H AEPySD Average distance of hind AEP to the center of the body, STD y axis

AR F PEPySD Average distance of front PEP to the center of the body, STD y axis

AS H PEPySD Average distance of hind PEP to the center of the body, STD y axis

AT F AEPx Average position of front AEP to the center of the body, x axis

AU H AEPx Average distance of hind AEP to the center of the body, x axis

AV F PEPx Average distance of front PEP to the center of the body, x axis

AW H PEPx Average distance of hind PEP to the center of the body, x axis

AX F AEPxSD Average position of front AEP to the center of the body, STD x axis

AY H AEPxSD Average distance of hind AEP to the center of the body, STD x axis

AZ F PEPxSD Average distance of front PEP to the center of the body, STD x axis

BA H PEPxSD Average distance of hind PEP to the center of the body, STD x axis

BB 3 leg swing Three leg swing index (Canter)

BC Speed SD Instantaneous speed standard deviation

BD framesON Number of frames where the animal is being detected

BE body stbl Stance linearity but for the body line, instead of the stance trace.

BF F_AEP STD Overall STD for front AEP, combines 2 feet and xy component

BG H_AEP STD Overall STD for hind AEP, combines 2 feet and xy component

BH F_PEP STD Overall STD for front PEP, combines 2 feet and xy component

BI H_PEP STD Overall STD for hind PEP, combines 2 feet and xy component

BJ SwingV STD Swing speed STD

BK all swing All swing index (jump/run)

BL Max Mdist Average of maximum Hind (L/R) opening (x component)

BM swT+stT Period (swing + stance time)

BN duty Fact Duty factor (Stance time/period)

BO Phase F Average phase for all front step cycles

BP Phase H Average phase for all hind step cycles

BQ areaLF Total LF footprint area

BR areaLH Total LH footprint area

BS areaRF Total RF footprint area

BT areaRH Total RH footprint area

BU pressLF Total LF pressure area (pixel intensity/area)

BV pressLH Total LH pressure area

BW pressRF Total RF pressure area

BX pressRH Total RH pressure area

BY fps Frame rate (fps)

BZ px/mm Calibration value in pixels/mm

CA stnc lin SD STD for stance linearity for all legs

CB SD AEP SD STD for the average AEP footprint clustering for all legs

CC SD PEP SD STD for the average PEP footprint clustering for all legs

CD DutyF SD STD for the Duty factor for all legs
