# Supplementary material for: Quantification of gait parameters in freely walking rodents
Source: BMC Biol. 2015 Jul 22;13:50. doi: 10.1186/s12915-015-0154-0 (PMC4511453; doi:10.1186/s12915-015-0154-0)
Supplement: Additional file 14: Table S2. — Videos of walking mice with a duty factor higher then 0.5 correspond to a walk-like behavior are highlighted in green. Videos with a duty factor higher then 0.5 correspond to a run-like behavior are highlighted in red. With the exception of one recording, all running animals display frames with an areal phase (underscored). [file 12915_2015_154_MOESM14_ESM.docx]

Supplemental Table 2

| *speed* |  | *duty factor* | *all swing index* |
| --- | --- | --- | --- |
| 23.4 | *walk* | 0.609 | 0.000 |
| 24.8 |  | 0.597 | 0.000 |
| 40.8 |  | 0.579 | 0.000 |
| 37.8 |  | 0.562 | 0.000 |
| 47.4 |  | 0.546 | 0.000 |
| 38.7 |  | 0.540 | 0.000 |
| 40.5 |  | 0.539 | 0.000 |
| 34.7 |  | 0.520 | 0.000 |
| 63.5 |  | 0.517 | 0.000 |
| 43.2 |  | 0.501 | 0.000 |
| 43.8 | *run* | 0.493 | 0.011 |
| 53.7 |  | 0.490 | 0.007 |
| 49.2 |  | 0.476 | 0.006 |
| 52.3 |  | 0.471 | 0.000 |
| 59.3 |  | 0.459 | 0.034 |
| 72.9 |  | 0.449 | 0.056 |

Videos of walking mice with a duty factor higher then 0.5 correspond to a walk-like behavior are highlighted in green. Videos with a duty factor higher then 0.5 correspond to a run-like behavior are highlighted in red. With the exception of one recording, all running animals display frames with an areal phase (underscored).
